# First neurotranscriptome of adults Tambaquis (*Colossoma macropomum*) with characterization and differential expression between males and females

**DOI:** 10.1038/s41598-024-53734-5

**Published:** 2024-02-07

**Authors:** Josy Miranda, Ivana Veneza, Charles Ferreira, Paula Santana, Italo Lutz, Carolina Furtado, Patrick Pereira, Luan Rabelo, Cristovam Guerreiro-Diniz, Mauro Melo, Iracilda Sampaio, Marcelo Vallinoto, Grazielle Evangelista-Gomes

**Affiliations:** 1 Laboratório de Genética Aplicada, Instituto de Estudos Costeiros, Universidade Federal Do Pará, Al. Leandro Ribeiro S/N – Bairro Aldeia, Bragança, Pará ZIP Code: 68600-000 Brazil; 2 Universidade Federal do Oeste do Pará, Campus Monte Alegre, Av. Major Francisco Mariano – Bairro Cidade Alta, Monte Alegre, Pará ZIP Code 68220-000 Brazil; 3 Divisão de Genética, Instituto Nacional de Câncer José de Alencar Gomes da Silva (INCA), Pr. da Cruz Vermelha, 23 – Bairro Centro, Rio de Janeiro, ZIP Code: 20230-130 Brazil; 4 Laboratório de Biologia Molecular e Neuroecologia, Instituto Federal de Educação, Ciência E Tecnologia Do Pará, - Campus Bragança, Rua da Escola Agrícola S/N – Bairro Vila Sinhá – Caixa Postal 72, Bragança, PA ZIP Code: 68600-000 Brazil; 5 Laboratório de Evolução, Instituto de Estudos Costeiros, Universidade Federal do Pará, Al. Leandro Ribeiro S/N – Bairro Aldeia, Bragança, Pará ZIP Code: 68600-000 Brazil; 6 Laboratório de Genética e Biologia Molecular, Instituto de Estudos Costeiros, Universidade Federal Do Pará, Al. Leandro Ribeiro S/N – Bairro Aldeia, Bragança, Pará ZIP Code: 68600-000 Brazil

**Keywords:** Genetics, Functional genomics, Genetic markers, Genomics

## Abstract

The Tambaqui is one of the most representative Amazon fish species, being highly exploited in fisheries, aquaculture and as a research model. Nonetheless, data about functional genome are still required to evaluate reproductive and nutrition parameters as well as resistance to pathogens. The of next-generation sequencing has allows assessing the transcriptional processes in non-model species by providing comprehensive gene collections to be used as a database in further genomic applications and increased performance of captive populations. In this study, we relied on RNAseq approach to generate the first transcriptome of the telencephalon from adult males and females of *Colossoma macropomum*, resulting in a reference dataset for future functional studies. We retrieved 896,238 transcripts, including the identification of 267,785 contigs and 203,790 genes. From this total, 91 transcripts were differentially expressed, being 63 and 28 of them positively regulated for females and males, respectively. The functional annotation resulted in a library of 40 candidate genes for females and 20 for males. The functional enrichment classes comprised reproductive processes (GO:0,048,609; GO:0,003,006; GO:0,044,703; GO:0,032,504; GO:0,019,953) being related to sex differentiation (e.g., SAFB) and immune response (e.g., SLC2A6, AHNAK, NLRC3, NLRP3 and IgC MHC I alpha3), thus indicating that the genes in the neurotranscriptome of Tambaqui participate in sex differentiation and homeostasis of captive specimens. These data are useful to design the selection of genes related to sex determination and animal welfare in raising systems of Tambaqui.

## Introduction

The Tambaqui is the most produced native fish in Brazilian aquaculture^1^ because of some favorable traits, such as tolerance to hypoxic waters, resistance to pathogens, easy production of fingerlings and rapid growth in captivity^2^. Despite this, there is still huge potential to expand its production^1^. Genomic studies represent potential strategies to improve aquaculture systems since the data generated by advanced sequencing and bioinformatic approaches can be successfully used in selection and breeding programs^3–5^

In fact, the evaluation of genes that play a key role in the metabolism of several fish species, including *Colossoma macropomum*, has been already carried out. These reports provide a database for advanced research to be tested in improvement of fish culture^6,7^. For instance, functional genome information based on transcriptome of distinct tissues from Tambaqui is available, representing a promising tool for the development of captive stocks, such as evaluation of expressed genes in specimens exposed to pathogens and distinct climate conditions or genetic pathways related to breeding, nutrition and physical traits^8–13^.

A diversified transcriptome dataset is essential to the full understanding of animal physiology inasmuch as the gene expression will vary according to the analyzed tissue, development stage, sex and environmental conditions of each species^14^. In particular, brain tissue has been widely studied in fishes since the neurotranscriptome analyses has provided several insights about behavioral patterns. Nonetheless, little is known about the genes expressed in distinct areas of encephalon in Tambaqui. A report by^10^ analyzed the transcriptome of telencephalon of Tambaqui raised under distinct conditions of physical activity, revealing that specimens exposed to exercises were characterized by an increased number of positively regulated genes related to memory and learning as well as faster growth. However, this transcriptome was obtained from juvenile specimens while data about adult and sexually mature fish remain unpublished.

**Figure 1:**
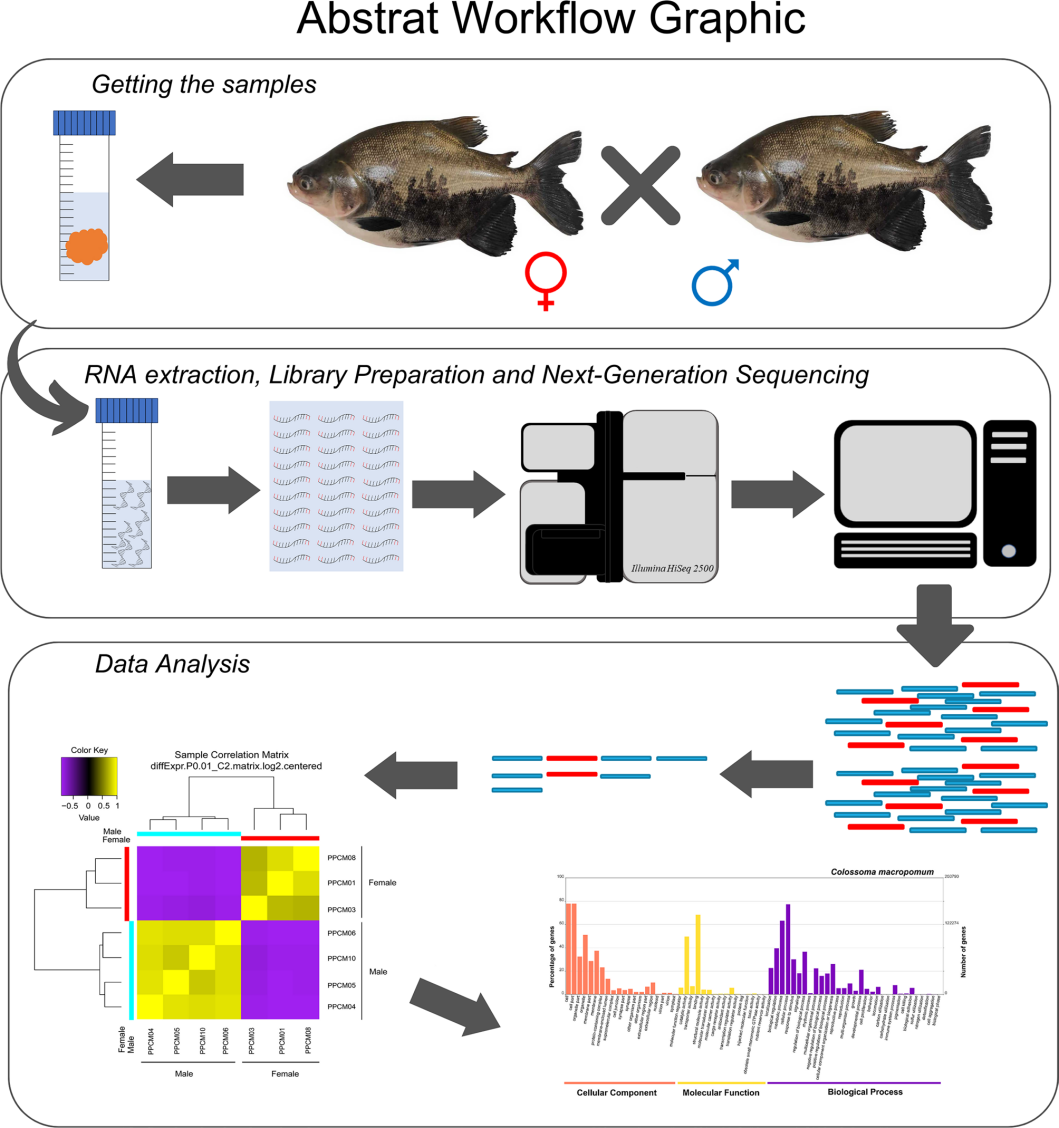
Graphical summary, showing the main steps for generating and analyzing data for this work, including tissue collection from male and female individuals, sample processing, experiments and analysis.

A variety of hormones are regulated by neuroendocrine signals that exhibit complex reaction cascades, like sex steroids, neuroendocrine-immune interactions, and stress response^15–19^. These mechanisms may play different roles in organisms and may be associated with other unclear functions in Tambaqui. Furthermore, the captive specimens are exposed to stressful environments distinct from those found in wild. Even after replicating natural conditions such as water currents, the natural breeding of Tambaqui has not been accomplished in captivity and positive regulation of genes related to stress stimuli has been reported^10^. Therefore, taking into account the importance of Tambaqui in aquaculture and the reliability of next generation molecular data in analyses of functional genomics, the present study provided useful information for further research by generating the first functional annotation of differentially expressed genes in the Neurotranscriptoma of adult males and females of *C. macropomum*. A graphical summary is available in Fig. 1, showing the main steps.

## Results

### Characterization of the dataset

The seven sequenced libraries of Tambaqui telencephalon comprised from 245,166.54 to 707,867.80 reads, with a mean value of 13,903,652.29 per library. After trimming, these values ranged from 229,145.61 to 681,344.40 reads with a mean GC content of 44% and length between 10 and 149 bp (Table 1).

**Table 1:** RNA-seq data for the sequenced samples, including information about the rate of reads per library and their main features. In gene expression analyses, we employed normalized values based on the Trimmed Mean of M-values (TMM) method.

Sample	Total Sequences	Trimmed sequences	Sequence length*	%GC*
PPCM01	15,660,140.00	13,115,353.00	10–149	45
PPCM03	24,516,654.00	22,914,561.00	10–149	44
PPCM08	19,262,065.00	18,372,066.00	10–149	44
PPCM04	13,000,078.00	10,366,722.00	10–149	43
PPCM05	12,682,833.00	12,374,601.00	10–149	43
PPCM06	7,078,678.00	6,813,444.00	10–149	45
PPCM10	15,249,815.00	13,368,819.00	10–149	42
All	107,450,263.00	9,325,566.00		

*after trimming.

The compiled libraries generated a total of 107,450,263.00 raw sequences that after trimming were reduced to 97,325,566.00 reads with an overall alignment rate of 97.71%. These values and other measurements of this dataset are shown in Table 3. The assembled reads using de novo method retrieved a transcriptome composed of 896,238 transcripts and 267,785 contigs, including isoforms (Table 2) and N50 values described in Supplementary Material 1.

**Table 2:** Statistics of raw data, trimmed data, original assembly, and transcriptome assembly of telencephalon transcriptome from *C. macropomum*.

Raw data	
Number of raw reads	107,450,263
Total Bases (bp)	83,1395 MB
Average GC content	44%
Trimmed data	
Number of reads after trimming	97,325,566
No concordantly aligned reads	28,379,831 (29.16%)
1-time concordantly aligned reads	28,659,692 (29.45%)
> 1-time concordantly aligned reads	40,286,043 (41.39%)
No concordantly aligns read pairs:	28,379,831
1-time discordantly aligned read pairs	9,346,861 (32.93%)
No aligned read pairs	19,032,970
Concordantly read pairs:	38,065,940
Not aligned	4,451,474 (11.69%)
1-time aligned	1,416,573 (3.72%)
> 1-time aligned	32,197,893 (84.58%)
Overall alignment rate	97.71%
De novo assembly	
Number of transcripts	896,238
Number of contigs	267,785

### Functional annotation of transcriptome

**Figure 2:**
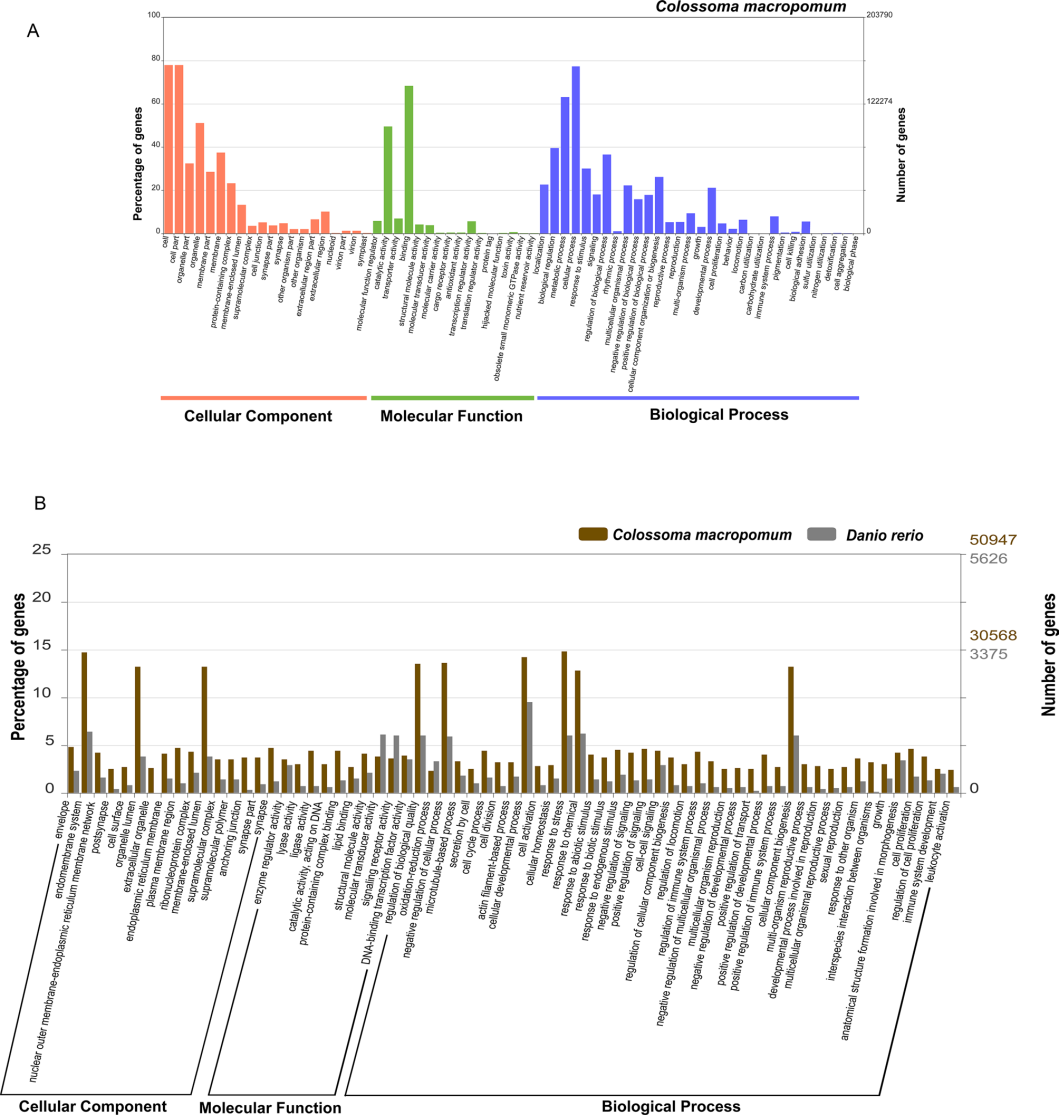
WEGO functional annotation output. Number and percentage of genes and GO terms in the Neurotranscriptoma of Tambaqui and a comparative analysis with the zebrafish Neurotranscriptoma.

In order to provide an enhanced description of the Tambaqui transcriptome, the 267,785 contigs were compared to the Swiss-Prot and RefSeq datasets. As a result, we retrieved 203,790.00 significant equivalent sequences (76.10%) related to 67 GO terms (Fig. 2A), totaling 533,155 putative genes distributed among the three Gene Ontology (GO) classes. When comparing the ontology terms of the Tambaqui with those of the zebra fish, we obtained 50,947 and 30,568 respectively (Fig. 2B).

Most of GO terms were related to Molecular Function (MF) (178,971), encompassing 16 terms, being followed by Biological Process (BP) (178,545 distributed into 31 categories), and Cellular Component (CC) (175,639) corresponding to 20 GO terms (Table 3).

**Table 3:** Description of functional annotation in the transcriptome of Tambaqui, showing the number of annotated genes and the GO term generated using WEGO 2.0.

		Tambaqui	Zebrafish	Total
Annotated Genes		203,790	22,504	226,294
GO Terms	Biological	178,545	15,503	194,048
	Cellular	175,639	15,116	190,755
	Function	178,971	16,150	195,121
	Total	533,155	46,769	579,924

**Figure 3:**
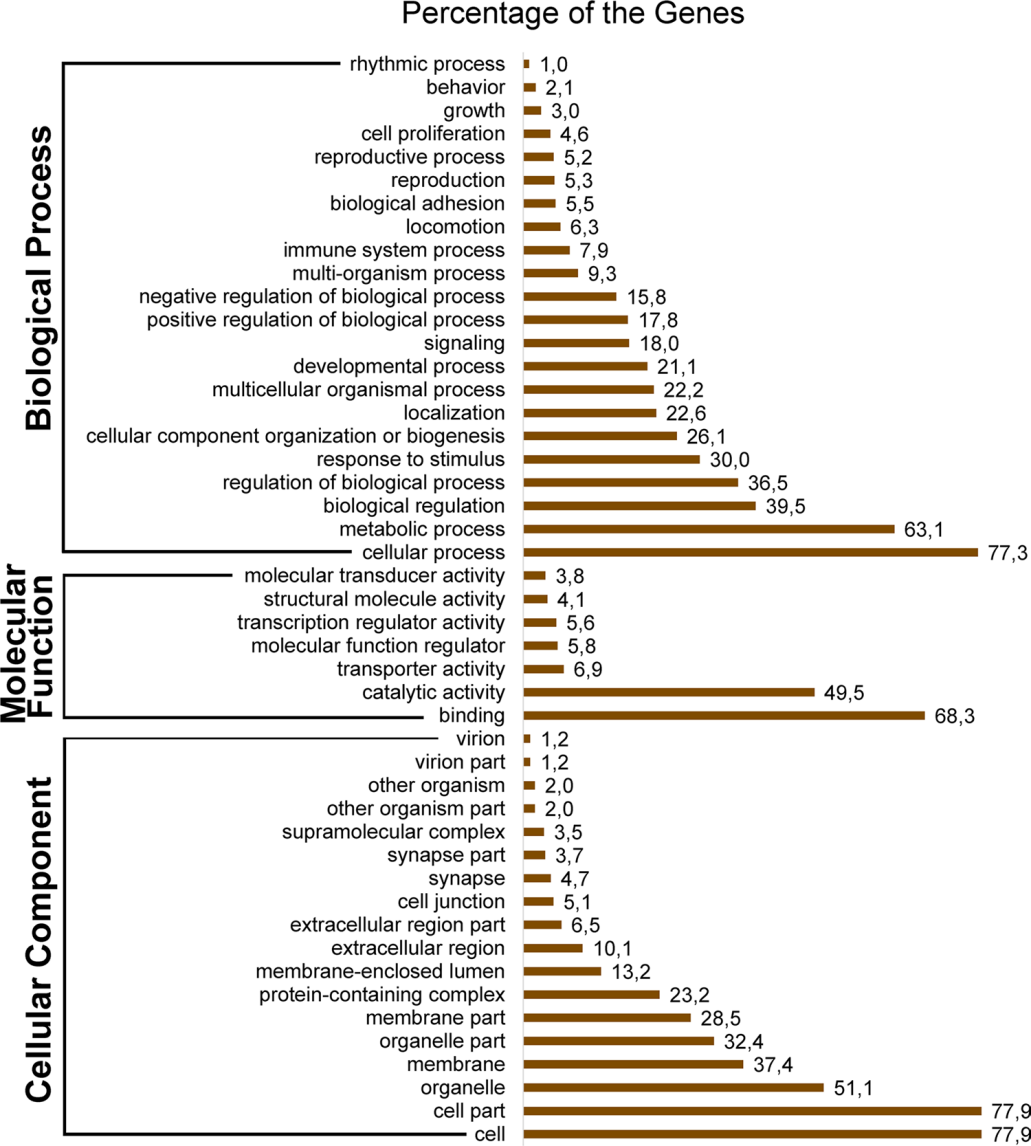
Classes of GO Terms. Classification and percentage of genes annotated in the Tambaqui Neurotranscriptoma and their distribution according to Biological Processes (BP), Molecular Function (MF) and Cellular Components (CC). Values > 1.

The classes with the highest correspondence within BP were the cellular processes (77.3%), metabolic processes (63.1%) and biological regulation (39.5%). As for the CC class, the cell (77.9%), cell part (77.9%) and organelles (32.4%) were the most representative GO terms. In turn, most of terms within MF were related to binding (68.3%), catalytic activity (49.5%) and transport activity (6.9%) (Fig. 3).

Taking into account the BP class, the following GO terms should be pointed out: regulation of immune system processes (GO:0,002,682), response to stimuli (GO:0,009,719, GO:0,009,607, GO:0,009,628), response to stress (GO:0,006,950), development of the immune system (GO:0,002,520), reproductive processes in multicellular organisms (GO:0,048,609), development of processes involved in breeding (GO:0,003,006), reproductive processes in multicellular organisms (GO:0,044,703), reproduction of multicellular organisms (GO:0,032,504) and sexual reproduction (GO:0,019,953).

### Analysis of differential expression

**Figure 4:**
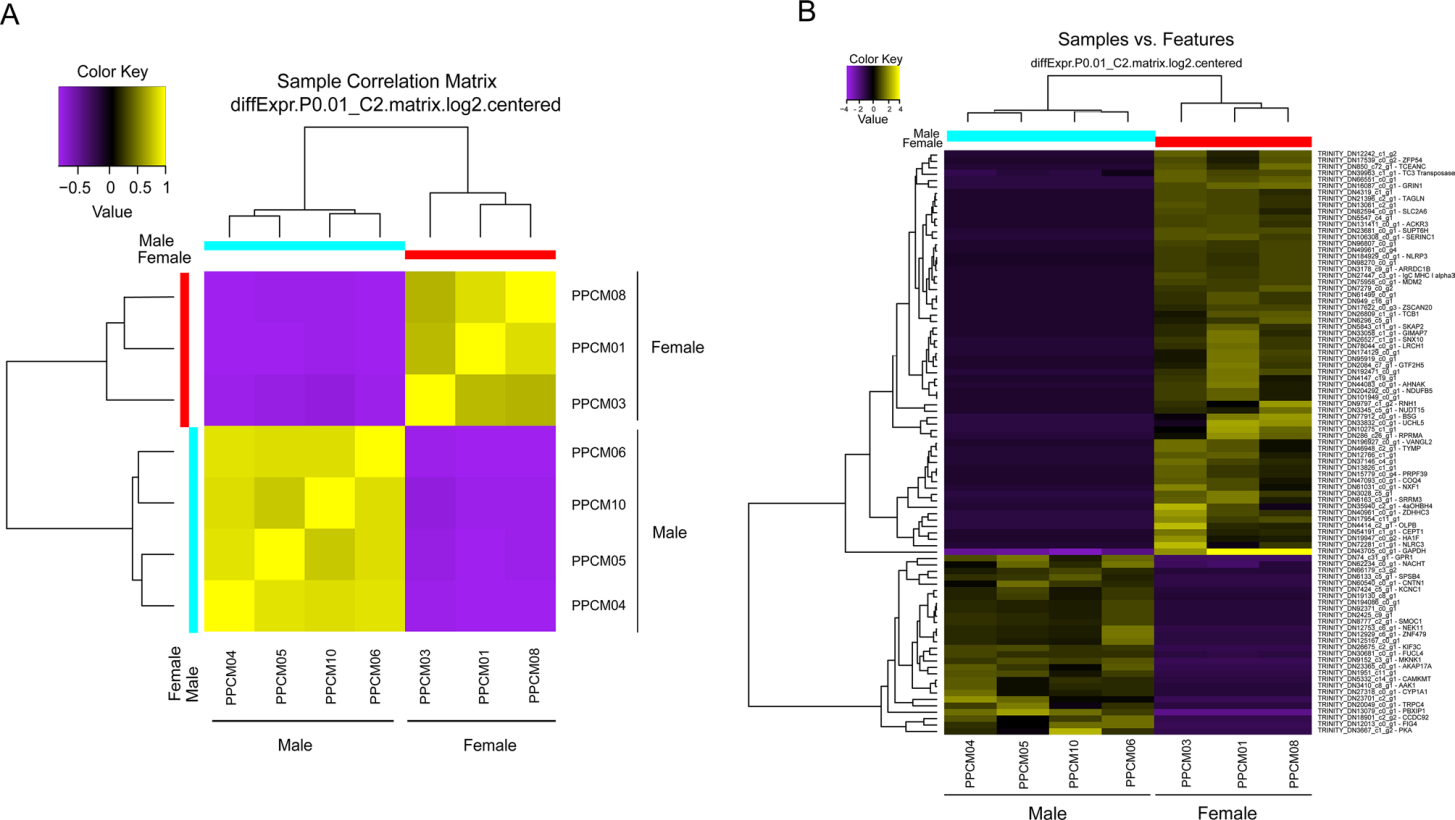
Hierarchical clusters based on TMM values. (**A**): Sample correlation matrix of hierarchically grouped samples after comparing the transcription expression values for each pair of samples (*P* < 0.01). (**B**): Heatmap showing the differentially expressed genes (male and female) of *C. macropomum* (*P* < 0.01).

The correlation matrix taking into account the 267,785.00 contigs revealed significant clusters among individuals, separating males and females into two distinct closely related groups (Fig. 4A). When the relative expression levels of each transcript in each sample was compared between male and female clusters, we identified 91 differentially expressed transcripts. The results based on heatmap analyses showed that the transcripts of each sex form separate clades, including 63 upregulated transcripts for females and 28 transcripts related to the males of *C. macropomum* (Fig. 4B). Supplementary materials 2:

The expression patterns in transcripts were significantly different between males and females. A total of 61 out of the 63 transcripts (96.82%) were expressed in females (reaching expression values of up to 20,107) but not in males. Even though two transcripts were shared between both groups, their expression values were highly divergent, ranging from 30,170 to 269,017 in females and from 0.000 to 9,257 in males (Supplementary materials 3: Table S1).

In turn, 26 out of the 28 upregulated sequences in males (92.85%) were expressed exclusively in this group, with expression values varying from 0.816 to 38,412. Two of these transcripts were also expressed in females but at extremely low levels (0.000 to 2,025) while they were overexpressed in males (values between 3,385 and 27,220) (Supplementary materials 3: Table S1).

### Functional annotation of DEGs in males and females of Tambaqui

**Figure 5:**
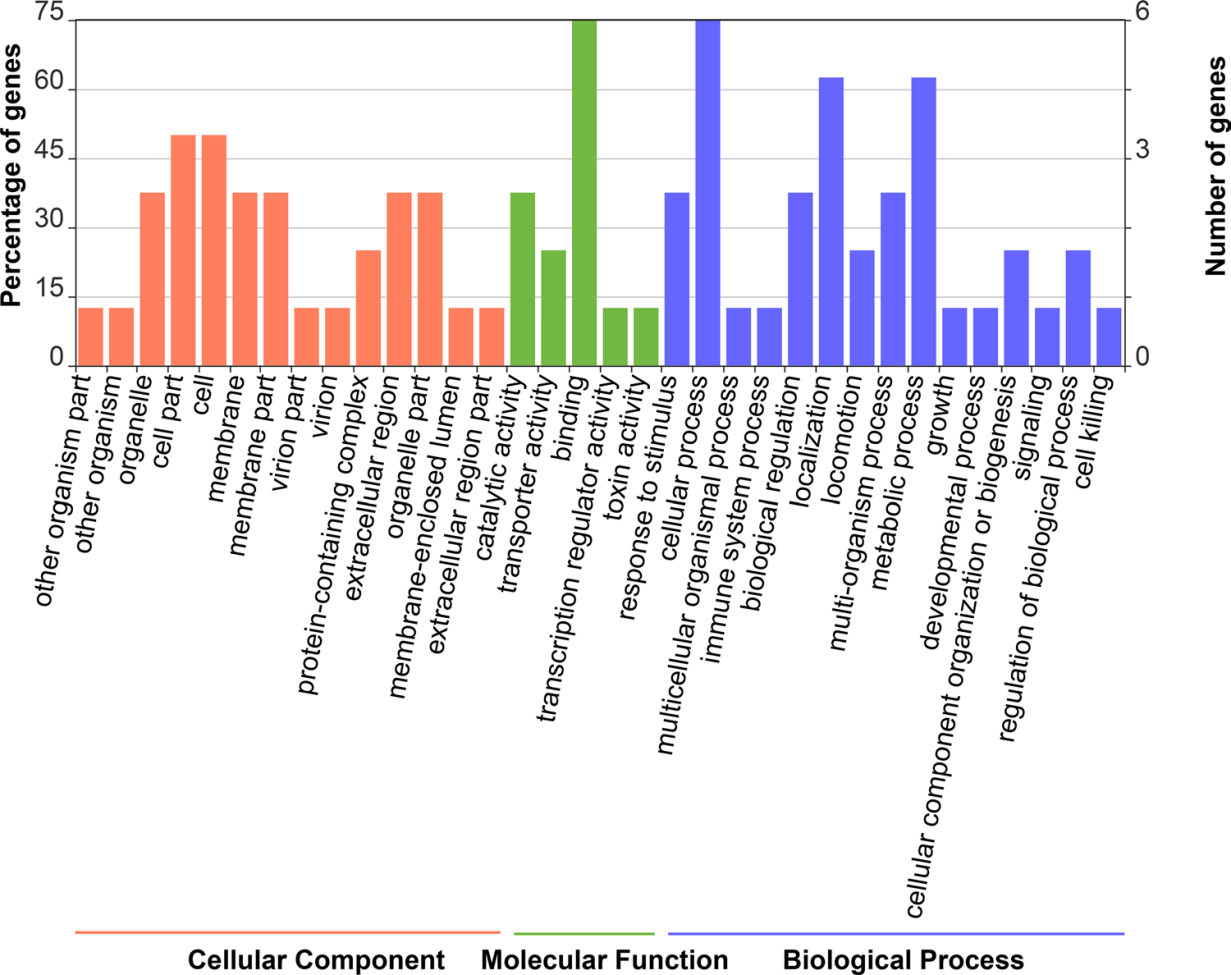
Functional annotation. of differentially expressed genes (DEGs.). Frequency and abundant GO terms under biological process, molecular function and cellular component categories in the DEGs.

To obtain a detailed analysis of DEGs, we evaluated the functional annotation of DEGs separately, resulting in 34 GO classes corresponding to Biological Processes (14), Molecular Function (5) and Cellular Component (15) (Fig. 5). The most representative GOs in MF were binding, catalytic activity and transport activity. As for the CC classes, the most relevant GO terms were cell, cell part and organelles. In for BP, cellular process, localization and metabolic processes were the predominant terms. We also highlight the role of putative genes related to reproduction, immune system and response to stimuli.

Considering the abovementioned GO terms, we added the description of 60 novel candidate genes. The females represented the group with the highest number of transcripts corresponding to putative genes, totaling 40 equivalences from the 63 upregulated transcripts. On the other hand, 20 matches were reported among the 28 upregulated transcripts of males (Supplementary materials 4: Table S2). Additional information about the functional annotation based on Blast2Go is available in Supplementary materials 5: Table S3.

The candidate genes identified in the present study play distinct roles in several and important biological pathways. For instance, SLC2A6, AHNAK, NLRC3 and IgC MHC I alpha3 are involved in inborn immunity, response to inflammation and other immune processes regulated by inflammatory stimuli. After a refined manual inspection of the present dataset, we identified upregulated (TRINITY_DN286_c26_g1) sequences in females, characterized by a similarity of 95% with the XM_017693343.2 sequence from *Pygocentrus nattereri* via NCBI BlastX. This sequence corresponds to the Factor B for Scaffold Attachment (SAFB), a gene that plays a key role as a co-repressor of alpha receptors of estrogen, an important female sex hormone.

## Discussion

### Transcriptome as source of information

The RNASeq method provides a wide coverage of non-repetitive genes and their isoforms, thus allowing accurate measures of expression profiles in transcriptome and analyses of phenotypic variation among individuals^14,20–22^. In fact, these sequences have been added to public databases worldwide being particularly helpful to describe and predict the role of thousands of proteins that take part in major biological pathways. Therefore, novel data about these genes are essential to relate the retrieved sequences to their putative functions^4,23^.

In addition, the sequencing method and the experimental design might directly influence the amount and the quality of retrieved reads^24^. In the present study, a significant number of reads with adequate coverage was generated inasmuch as libraries composed of up to 24,516,654 reads were obtained, as well as an assembled transcriptome characterized by 97% of aligned sequences and a high number of annotated genes (203.790). These results indicate the Illumina platform and the sampling strategy was successful to the goals of this work, thus determining satisfactory levels of functional annotation.

Actually^25^, reported that Illumina MiSeq presented some advantages when compared to other available platforms at that time, particular in relation to the quality parameters. Nonetheless, the Illumina platform demands a high computational effort to analyses of large datasets^24^. Therefore, the development of additional methods that refine these data by reducing the number of sequences used in gene annotation, such as differential expression approaches, has been important to generate fast discrimination of experimental groups^26^.

### Differential expression: males and females

This is the first transcriptome generated from the telencephalon of adult specimens of Tambaqui. As a portion of the central nervous system, the telencephalon which participates in several mechanisms linked to the emotional state and endocrine system of fishes, including reproduction and animal homeostasis^27^.

The transcripts expressed in the neurotranscriptome of Tambaqui generated a divergent pattern between males and females, revealing the importance of telencephalon as a major source of information to differential gene expression between sexes^28^. On average, 98.8% of transcripts were overexpressed exclusively in one group but not in the other, while females were characterized by a larger number of differentially expressed genes or DEGs (63). In contrast^29^, suggested that upregulated transcripts tend to be more numerous in brain of males, thus differing from our results. Even though these authors analyzed a similar number of libraries when compared to the present work, most values referred to the male brain library (86,112,076 reads) what could lead to a biased amount of DEGs in male tissue. Furthermore, the increased amount of DEGs in females of Tambaqui reported in our study represents an important landmark to investigate genes related to sex determination.

The identification of transcripts related to major reproductive pathways in the neurotranscriptome of Tambaqui indicates that the brain of this species might encompass additional information that can be used to elucidate the mechanisms of sex differentiation in *C. macropomum*. Therefore, further studies are recommended to seek sex-related genes beyond those related to DEGs, which is accomplishable even for non-model organisms. For example^29^, successfully annotated 82% and 90% of upregulated transcripts in the brain transcriptome of females and males of tropical gar, respectively.

Several research related to the early differentiation between males and females have been carried out in farm animals, particularly related to increase market gains, since a specific sex might be more advantageous than the other in animal production, as the masculinization of Tilapia females to increase the fish weight^30^. In the case of Tambaqui, the females present fast growth rates, with weight gains up to 16% higher than those reported in males, making the females more attractive for the production in captivity [31; 32].

In our study, females weighed 1.4 kg, 1.5 kg and 2.6 kg, and were most likely at the same stage of gonadal development, since females of this species, when bred in captivity, present late gonadal development compared to males, starting with approximately 1,200 kg, and do not complete their maturation before 3 kg, which contributes to these showing greater growth^31^.

The mechanisms involved in the reproductive process and sex determination of fishes are particularly diversified, unlike other groups of vertebrates, such as mammals. These aspects are not phylogenetically conserved and distinct strategies have evolved even in closely related species. Such plasticity of sex differentiation in teleosteans is directly related to sex steroid hormones, possibly regulated by specific genes expressed in brain networks^18,28^.

The Tambaqui is a rheophilic fish, therefore, environmental stimuli are extremely important for their reproduction. When these fish are cultivated in the northern region of Brazil, males can reach sexual maturity at any time of the year, however, it is believed that the lack of environmental stimuli inhibits the production of progestin, or produce little, which prevents spawning from occurring in captivity^31^.

Accordingly, GO terms associated with fish reproduction (GO:0,048,609; GO:0,003,006; GO:0,044,703; GO:0,032,504; GO:0,019,953) were presently observed among the metabolic pathways in the neurotranscriptome of Tambaqui. The presence of these transcripts is expected since major hormones are produced and/or activated in the hypothalamus. Some of these genes have already been reported in zebrafish, a model species, like arginine vasotocin receptors^33^ and gonadotropin-releasing hormone 3 receptor^34^, but these and some other genes were not differentially expressed in Tambaqui neurotranscriptome.

Most likely, this evidence is explained by the fact that the Tambaqui is a non-model organism in spite of their economic importance and hence the lack of information about this species hindered a more precise functional annotation. Recently^13^, identified that cyp19a1a and cyp19a1b genes would play a key role in the differentiation of males, but the genes related to similar processes in females are largely unknown. In the present study, high gene expression values were observed for the SAFB in females (11,496; 1,526; 5,939) while it remained unexpressed in males. The SAFB is highly expressed in brain tissues, encoding a co-repressor of estrogen receptors that interact with alpha receptors (ERα). In mice, the overexpression of SAFB in brain was associated with a normal distribution of gonadal steroid hormones at specific development stages, besides playing a putative role in the establishment of genitalia, formation of dimorphic brain structures and reproductive sex behavior^35^.

Therefore, the presence of SAFB in females of Tambaqui is a strong indicator that this gene is somehow related to the sex differentiation of females. The estrogen receptor (ER) exhibits a close relationship with 17β-estradiol, a hormone used in the feminization process of captive Tambaqui, which is co-expressed along with SAFB and induces significant changes in sex differentiation^36^. The Tambaqui is a rheophilic fish in which the release of steroid hormones and spawning are related to upstream migration under normal conditions in wild. The presence of overexpressed SAFB co-repressor in captive females may be a key feature that prevents the natural female maturation in culture systems, since maturity has only been achieved in females fed on diets rich in 17β-estradiol^36^.

### Stress- and immune-related genes

In fish, the hypothalamic-pituitary-interrenal (HPI) system participates in the mechanisms of response to stress by promoting a cascade of reactions that triggers the release of adrenocorticotropin (ACTH), thus stimulating the production of cortisol to assist in the regulation of the homeostasis of both organs and tissues^37^. As a matter of fact, the functional annotation in the present study revealed several GO terms in the Biological Processes associated with the responses to stress and stimuli. Captive fishes are particularly susceptible to adverse conditions such as hypoxia, high stock densities and poor water quality during transportation, translocation to fish tanks, changes in diets during growth among others^38–40^.

In addition, fish farms are usually established close to large animal culture systems thus being potentially affected by pollution and contamination from these systems eventually leading to effects on physiological and biochemical parameters of captive fish stocks^41^. Because of these impacts, studies focused on animal welfare have increased over the last years and thus understanding the genetic mechanisms underlying these pathways is extremely relevant.

The changes of environmental conditions in captivity are stressful to aquatic organisms, thus activating their defense immune responses^42^. In fact, analyses of neurotranscriptome have already been useful to identify stress mechanisms in fishes^43–45^. The functional annotation in the present study identified the following genes related to fish immune system: NLRP (3 and 12) and IgC MHC I alpha3.

Two domains (3 and 12) of the NLRP gene were annotated in this work. This gene has already been widely reported in mammalian species it has been expressed in fish samples exposed to infectious agents, such as in the Japanese sole after bacterial infection^46^, and in the mucus of *Seriola dumerili* parasitized by *Neobenedenia girlellae*^47^. Similarly, the upregulated expression of IgC MHC I alpha 3 has also been related to exposure of fishes to pathogens, as reported in the transcriptome of specimens of *Salmo salar* after viral infection^48^. Thus, the expression of these genes in the neurotranscriptome of Tambaqui suggests a metabolic response of captive specimens to microorganisms from their environment in order to maintain animal homeostasis.

Even though this research was focused on the characterization of expression profiles, these results provide a baseline for further studies to the development of control markers related to animal welfare. This approach might be useful to direct proper strategies against parasites, contaminants and stressful agents that could jeopardize the animal growth.

The goal of this study was to assemble, predict and discuss the differential expression in transcripts of the neurotranscriptome in adult Tambaqui in order to provide a database that can guide further sex-related gene selection strategies. The neurotranscriptome herein reported encompassed 91 differentially expressed genes related to major ontology classes, such as reproductive process, stress response and immune system. In particular, an upregulated SAFB gene was identified in females, was identified, which putatively plays a key role in the sex differentiation of females, as well as candidate genes related to the immune system, such as the NLRP (3 and 12) and the IgC MHC I alpha3. In future, additional research focusing on detailing the role of these genes can contribute to the development of improved practices in the production of captive Tambaqui such as the formation of feminized stocks and reduced environmental stress in culture systems.

## Material and methods

### Ethics statement and sampling

The specimens used in this study were collected and transported according to the license granted by Instituto Chico Mendes da Biodiversidade (ICMBio, n. 60,833–1). Prior to the collection of biological material, the animals were anesthetized by immersion in a solution containing a concentration of 200 mg/L of benzocaine and water^49^ and slaughtered by section of spinal cord and major blood vessels according to the procedures approved by the National Counsel of Animal Experimentation Animal (CONCEA, law 11.794) and by the Committee of Experimentation and Use of Animals (CEUA, n. 7,399,260,721). We also declare that the experimental design and the entire study were carried considering the ARRIVE Guidelines (available at: https://arriveguidelines.org).

The specimens were collected during the rainy season (Amazonian winter). However, all were obtained from a commercial fish farm, where they were in controlled conditions of captivity. Therefore, seasonal variations that occur due to changes in seasons, they would not be affecting the physiology and metabolism of these fish, and consequently the animals' Transcriptome.

A portion of the telencephalon was selected for the present study. The tissues were obtained from seven adult (four males and three females) specimens of *C. macropomum* raised under the same management conditions in the fish station “Menino Deus”, municipality of Bonito, PA, northern Brazil (01°18′45.6″ S 047°21′51.3″ W). All specimens were weighed and measured (total length and total height) (Table 4). Besides the removal of telencephalon for RNA isolation, a muscle fragment was obtained from each fish for isolation of genomic DNA. The telencephalon samples were placed individually in 10-mL tubes containing RNA-preservative solution (RNA later® RNA Stabilization Solution, Ambion, Thermo Fisher, USA) and stored at − 20 °C. The muscle samples were stored in 2-mL Eppendorf tubes with 100% ethanol.

**Table 4:** Samples of adult specimens of *C. macropomum* collected in the fish station “Menino Deus”, municipality of Bonito–PA, including biometric data, storage conditions, isolated RNA information, library and barcode numbers.

Label	Species	Sex	Weight (kg)	Total length (cm)	Total height (cm)	Tissue storage		RNA concentration (ng/µl)	Amount (µl)	Library	Index
						Telencephalon	Muscle				
PPCM01	*C. macropomum*	Female	2.6	49	26	RNA Later	100% ethanol	150	50	1	B2
PPCM03	*C. macropomum*	Female	1.5	41.5	22	RNA Later	100% ethanol	90	60	2	C2
PPCM04	*C. macropomum*	Male	2.8	51	25	RNA Later	100% ethanol	270	50	3	D2
PPCM05	*C. macropomum*	Male	2.4	48	25	RNA Later	100% ethanol	150	80	4	E2
PPCM06	*C. macropomum*	Male	1.5	43	22	RNA Later	100% ethanol	120	80	5	F2
PPCM08	*C. macropomum*	Female	1.4	40	23	RNA Later	100% ethanol	75	50	6	G2
PPCM10	*C. macropomum*	Male	2.4	50	24	RNA Later	100% ethanol	120	90	7	H2

### Identification of pure and hybrid specimens

Total DNA was isolated from the muscle fragments using the Wizard Genomic DNA purification kit (Promega, Madison, WI, USA). The quality of the DNA was visualized via electrophoresis in 1% agarose gel, stained with blue juice and red gel. The DNA samples were submitted to multiplex PCR for identification of pure or hybrid specimens according to the band profile after amplification of the alpha-Tropomyosin nuclear gene, as described by^50^. The genotyping confirmed that all samples corresponded to pure specimens of *C. macropomum*.

### Isolation of RNA, construction of libraries and next-generation sequencing

The total RNA was isolated from 30 mg of from telencephalon tissue macerated in liquid nitrogen using the RNeasy Mini kit protocol (© QIAGEN). The quality and quantification (ng/µl) of RNA samples was evaluated in a Nanodrop 1000 (Thermo Scientific) and integrity in a 2100 Bioanalyzer Instrument (Table 4).

The RNA molecules were then fragmented and transcribed into cDNA. Individual libraries were constructed per cDNA sample, starting with 2000 ng of total RNA, using the SureSelect Strand Specific RNA Library Prep ILM Kit (Agilent Technologies) according to the manufacturer's protocol. In this process, unique barcodes for each sample and the adapters compatible with the sequencing platform were added, thus allowing the samples to be grouped and then individually identified (Table 4). Then, the libraries were transferred to the flow cell of the Illumina HiSeq 2500 platform, using the TruSeq SBS v3-HS kit. The procedures from RNA isolation to sequencing that resulted in raw sequence reads, paired-end: 2 × 100, of three female and four male replicates was carried out in the Genetic Center of the National Institute of Cancer José de Alencar Gomes da Silva (INCA) in Rio de Janeiro. The raw sequence reads were submitted to the Sequence Read Archive (SRA) database of the National Center for Biotechnology Information (NCBI) (accession code: PRJNA1016488).

### Pre-processing and de novo assembly

The raw sequences generated in Illumina platform were clustered and converted into FastQ files according to their index and barcode tags. The quality parameters of these sequences were analyzed individually in the software FastQC v 0.18^51^. Afterwards, the adapters, short or low-quality reads were discarded using the software Trimmomatic v. 0.40^52^, according to the trimming criteria reported by^53^ for non-model organisms.

After removing the low-quality reads, the contigs were assembled using de novo approach in default parameters of the software Trinity 2.13.2^54,55^. Since one of the major goals of this study was to analyze the differentially expressed genes (DEG) between males and females of Tambaqui, the de novo methodology is highly recommended^56^ since it allows identifying the most relevant DEG when compared to Ab initio approaches, even though a reference genome is already available for *C. macropomum*.

### Analysis of differential expression and functional annotation

Based on the assembled telencephalon transcriptome, we performed a comparative analysis by separating the biological replicates into two groups (males and females). The quantification of gene expression was carried out mapping back the assembled transcriptome to the trimmed RNA seq data through software Salmon 1.8.0^57^. The generated matrix of gene expressing obtained from the quantification step was used for the differential expression analysis using EdgeR v. 2.26.0^58^ implemented in R package v. 4.1.0 (2019), thus resulting in the identification of differentially expressed transcripts. For expression value normalization in this study, we employed the Trimmed Mean of M-values (TMM) method, which was also utilized for hierarchical cluster analysis. The selection of TMM was based on its ability to address variations in library size and composition, ensuring a more accurate representation of gene expression levels^55,59^.

Two strategies were used for the functional annotation of transcripts: (1) annotation of total transcriptome, and (2) annotation of DEGs. Therefore, the files were compared to distinct sequence datasets to provide the most complete coverage of functional annotation, starting from those available in UniProtKB/Swiss-Prot and UniProtKB/TrEMBL (Release 2022_11) and complemented by non-redundant databases from NCBI, Refseq protein, Uniref, GenPept, NCBI ntm and Refseq RNA (all accessed on November, 18, 2022), via Blast2GO^60^, considering E-values of 1e-3, and a minimum coverage of 90%. For sequences identified across multiple databases, we prioritized the best hit based on the identification criteria described earlier.

As for the DEGs, we carried out a manual inspection of hits. The unrecorded transcripts were manually annotated after searches in GeneCards (https://www.genecards.org) dataset to check the identified gene followed by search of similar gene sequences in AmiGO Gene Ontology^61^, using the database available in the model species *Danio rerio* (zebrafish) as reference. The official gene symbols were confirmed by the HUGO Gene Nomenclature Committee (HGNC) and NCBI gene database. The gene classes identified in Gene Ontology were then inserted in the annotation file.

At last, we related each transcript from both total transcriptome and DEGs to the each one of the three GO classes: BP, MF and CC (Gene Ontology Consortium). The resulting graphs were plotted using the online tool WEGO 2.0 available in https://wego.genomics.cn^62^. Using the same software, we also built a graph relating the annotation and the GO terms retrieved in Tambaqui to those reported in zebrafish to provide a comparative scenario between both fish species.

### Supplementary Information

Supplementary Legends.

Supplementary Information 2.

Supplementary Figure 1.

Supplementary Table S1.

Supplementary Table S2.

Supplementary Table S3.

## Data Availability

Accession codes: PRJNA1016488. Submitted data will be made available after publication. Reviewers can access it via the following link: https://dataview.ncbi.nlm.nih.gov/object/PRJNA1016488?reviewer=ac8607v0k50tqfdl4i4vlgvadt.
