# Supplementary material for: First neurotranscriptome of adults Tambaquis (Colossoma macropomum) with characterization and differential expression between males and females
Source: Sci Rep. 2024 Feb 7;14:3130. doi: 10.1038/s41598-024-53734-5 (PMC10850070; doi:10.1038/s41598-024-53734-5)
Supplement: Supplementary file 1 — Supplementary Legends. [file 41598_2024_53734_MOESM1_ESM.docx]

**Legend for Supplementary Material**

**Supplementary materials 1:** Dataset provides a comprehensive analysis of transcriptome assembly metrics. The workbook consists of three sheets: (1) Summary - offering a overview of gene and transcript data, (2) All Transcripts - detailing N50 statistics for all transcripts and contigs, and (3) Longest Isoform - focusing exclusively on N50 statistics for the longest isoform.

**Supplementary materials 2:** Volcano graphic representation of differentially expressed transcripts in male and female Tambaqui. In red are the most differentially expressed transcripts, the black dots correspond to the transcripts with the lowest differential expression values or no differential expression (-1*Log10FDR < 0.01).

**Supplementary materials 3:** TMM values of differential expression in the telencephalon transcriptome of adult males and females of blackfin pacu raised in captivity.

**Supplementary materials 4:** Functional annotation of differentially expressed transcripts in the neurotranscriptome of Tambaqui based on Blast2Go searches.

**Supplementary materials 5:** Comprehensive description of the functional annotation containing the sequence name, gene description, GO identities, GO names, protein family integrated resource IDs (InterPro) and InterPro Go names.
