# Supplementary figures and images for: First neurotranscriptome of adults Tambaquis (Colossoma macropomum) with characterization and differential expression between males and females
Source: Sci Rep. 2024 Feb 7;14:3130. doi: 10.1038/s41598-024-53734-5 (PMC10850070; doi:10.1038/s41598-024-53734-5)

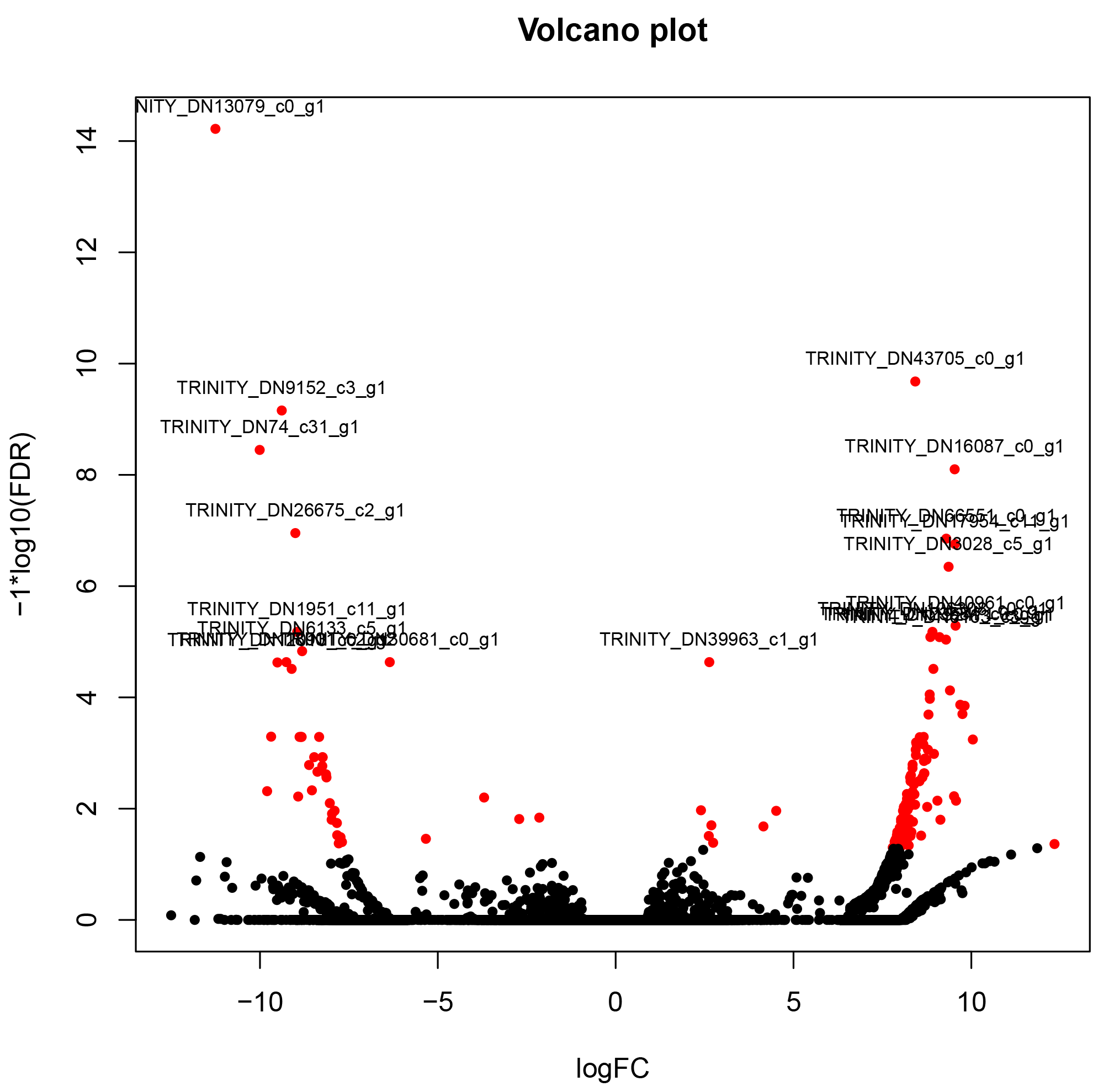

Supplement: Supplementary file 3 — Supplementary Figure 1. [file 41598_2024_53734_MOESM3_ESM.tif]
