# Supplementary material for: First neurotranscriptome of adults Tambaquis (Colossoma macropomum) with characterization and differential expression between males and females
Source: Sci Rep. 2024 Feb 7;14:3130. doi: 10.1038/s41598-024-53734-5 (PMC10850070; doi:10.1038/s41598-024-53734-5)
Supplement: Supplementary file 4 — Supplementary Table S1. [file 41598_2024_53734_MOESM4_ESM.docx]

**Table S1**: TMM values of differential expression in the telencephalon transcriptome of adult males and females of blackfin pacu raised in captivity.

| Samples | PPCM01 | PPCM03 | PPCM08 | PPCM10 | PPCM04 | PPCM05 | PPCM06 |
| --- | --- | --- | --- | --- | --- | --- | --- |
| Males | FPKM Values | | | | | | |
| TRINITY_DN8777_c2_g1 | 0.000 | 0.000 | 0.000 | 2.261 | 2.933 | 2.573 | 4.269 |
| TRINITY_DN1951_c11_g1 | 0.000 | 0.000 | 0.000 | 2.398 | 6.200 | 5.458 | 4.522 |
| TRINITY_DN3410_c8_g1 | 0.000 | 0.000 | 0.000 | 2.748 | 5.547 | 1.549 | 2.636 |
| TRINITY_DN13079_c0_g1 | 0.000 | 0.000 | 0.000 | 20.616 | 20.984 | 38.412 | 11.278 |
| TRINITY_DN60540_c0_g1 | 0.000 | 0.000 | 0.000 | 3.903 | 1.710 | 8.913 | 3.215 |
| TRINITY_DN12013_c0_g1 | 0.000 | 0.000 | 0.000 | 10.603 | 4.622 | 2.009 | 10.083 |
| TRINITY_DN5332_c14_g1 | 0.000 | 0.000 | 0.000 | 2.947 | 5.798 | 1.670 | 2.820 |
| TRINITY_DN3667_c1_g2 | 0.000 | 0.000 | 0.000 | 23.986 | 3.506 | 1.291 | 4.253 |
| TRINITY_DN27318_c0_g1 | 0.000 | 0.000 | 0.000 | 2.046 | 7.517 | 1.623 | 2.743 |
| TRINITY_DN74_c31_g1 | 0.000 | 0.000 | 0.000 | 4.194 | 8.186 | 14.152 | 11.906 |
| TRINITY_DN12929_c6_g1 | 0.000 | 0.000 | 0.000 | 2.351 | 3.043 | 2.675 | 8.869 |
| TRINITY_DN26675_c2_g1 | 0.000 | 0.000 | 0.000 | 4.047 | 5.116 | 5.622 | 4.423 |
| TRINITY_DN20049_c0_g1 | 0.000 | 0.000 | 0.000 | 0.816 | 4.001 | 10.292 | 3.205 |
| TRINITY_DN9152_c3_g1 | 0.000 | 0.000 | 0.000 | 5.859 | 5.099 | 6.660 | 7.406 |
| TRINITY_DN2425_c9_g1 | 0.000 | 0.000 | 0.000 | 2.097 | 2.729 | 2.387 | 3.969 |
| TRINITY_DN23701_c2_g1 | 0.000 | 0.000 | 0.000 | 2.328 | 16.802 | 9.602 | 2.246 |
| TRINITY_DN194086_c0_g1 | 0.000 | 0.000 | 0.000 | 1.987 | 2.593 | 2.263 | 3.766 |
| TRINITY_DN125167_c0_g1 | 0.000 | 0.000 | 0.000 | 2.022 | 2.636 | 2.303 | 7.663 |
| TRINITY_DN12753_c6_g1 | 0.000 | 0.000 | 0.000 | 2.400 | 3.100 | 2.729 | 9.044 |
| TRINITY_DN19130_c8_g1 | 0.000 | 0.000 | 0.000 | 1.475 | 1.960 | 3.390 | 2.859 |
| TRINITY_DN92371_c0_g1 | 0.000 | 0.000 | 0.000 | 1.953 | 2.550 | 2.223 | 3.703 |
| TRINITY_DN18901_c2_g2 | 0.000 | 0.000 | 0.000 | 4.976 | 6.518 | 1.884 | 9.485 |
| TRINITY_DN7424_c5_g1 | 0.000 | 0.000 | 0.000 | 1.767 | 2.311 | 4.019 | 3.361 |
| TRINITY_DN23365_c0_g1 | 0.000 | 0.000 | 0.000 | 1.888 | 7.321 | 4.294 | 7.164 |
| TRINITY_DN6133_c5_g1 | 0.000 | 0.000 | 0.000 | 6.534 | 3.483 | 3.951 | 2.891 |
| TRINITY_DN66179_c3_g2 | 0.000 | 0.000 | 0.000 | 3.851 | 1.974 | 2.908 | 2.473 |
| TRINITY_DN30681_c0_g1 | 0.000 | 0.052 | 0.062 | 3.385 | 4.773 | 3.541 | 3.587 |
| TRINITY_DN62234_c0_g1 | 0.756 | 0.966 | 2.025 | 10.079 | 5.907 | 23.090 | 27.220 |
| Females |  |  |  |  |  |  |  |
| TRINITY_DN49961_c0_g4 | 2.476 | 2.938 | 3.211 | 0.000 | 0.000 | 0.000 | 0.000 |
| TRINITY_DN98270_c0_g1 | 2.218 | 2.634 | 2.870 | 0.000 | 0.000 | 0.000 | 0.000 |
| TRINITY_DN101949_c0_g1 | 4.434 | 2.634 | 1.439 | 0.000 | 0.000 | 0.000 | 0.000 |
| TRINITY_DN17622_c0_g3 | 3.121 | 1.857 | 3.032 | 0.000 | 0.000 | 0.000 | 0.000 |
| TRINITY_DN33058_c1_g1 | 6.415 | 1.908 | 2.077 | 0.000 | 0.000 | 0.000 | 0.000 |
| TRINITY_DN7279_c0_g2 | 2.960 | 2.642 | 4.790 | 0.000 | 0.000 | 0.000 | 0.000 |
| TRINITY_DN2084_c7_g1 | 7.453 | 1.586 | 2.509 | 0.000 | 0.000 | 0.000 | 0.000 |
| TRINITY_DN16087_c0_g1 | 7.348 | 5.252 | 8.052 | 0.000 | 0.000 | 0.000 | 0.000 |
| TRINITY_DN21396_c2_g1 | 3.121 | 3.714 | 2.270 | 0.000 | 0.000 | 0.000 | 0.000 |
| TRINITY_DN61031_c0_g1 | 3.825 | 8.902 | 1.239 | 0.000 | 0.000 | 0.000 | 0.000 |
| TRINITY_DN78044_c0_g1 | 6.219 | 2.465 | 2.688 | 0.000 | 0.000 | 0.000 | 0.000 |
| TRINITY_DN17539_c0_g2 | 1.560 | 3.714 | 4.043 | 0.000 | 0.000 | 0.000 | 0.000 |
| TRINITY_DN6163_c3_g1 | 9.352 | 6.101 | 2.166 | 0.000 | 0.000 | 0.000 | 0.000 |
| TRINITY_DN949_c16_g1 | 3.651 | 2.173 | 2.362 | 0.000 | 0.000 | 0.000 | 0.000 |
| TRINITY_DN184929_c0_g1 | 2.218 | 2.634 | 2.878 | 0.000 | 0.000 | 0.000 | 0.000 |
| TRINITY_DN3178_c9_g1 | 2.341 | 2.779 | 3.037 | 0.000 | 0.000 | 0.000 | 0.000 |
| TRINITY_DN286_c26_g1 | 11.496 | 1.526 | 5.939 | 0.000 | 0.000 | 0.000 | 0.000 |
| TRINITY_DN33832_c0_g1 | 16.032 | 0.545 | 12.837 | 0.000 | 0.000 | 0.000 | 0.000 |
| TRINITY_DN12766_c1_g1 | 4.953 | 4.796 | 1.605 | 0.000 | 0.000 | 0.000 | 0.000 |
| TRINITY_DN4414_c2_g1 | 2.745 | 20.107 | 2.498 | 0.000 | 0.000 | 0.000 | 0.000 |
| TRINITY_DN131411_c0_g1 | 3.493 | 3.119 | 2.263 | 0.000 | 0.000 | 0.000 | 0.000 |
| TRINITY_DN17954_c11_g1 | 3.950 | 11.742 | 5.166 | 0.000 | 0.000 | 0.000 | 0.000 |
| TRINITY_DN40961_c0_g1 | 4.597 | 13.646 | 2.983 | 0.000 | 0.000 | 0.000 | 0.000 |
| TRINITY_DN6296_c5_g1 | 2.524 | 1.497 | 4.910 | 0.000 | 0.000 | 0.000 | 0.000 |
| TRINITY_DN75958_c0_g1 | 2.995 | 3.469 | 3.306 | 0.000 | 0.000 | 0.000 | 0.000 |
| TRINITY_DN3345_c5_g1 | 1.540 | 2.028 | 5.856 | 0.000 | 0.000 | 0.000 | 0.000 |
| TRINITY_DN44083_c0_g1 | 7.720 | 3.051 | 1.669 | 0.000 | 0.000 | 0.000 | 0.000 |
| TRINITY_DN9797_c1_g2 | 0.832 | 2.403 | 11.291 | 0.000 | 0.000 | 0.000 | 0.000 |
| TRINITY_DN12242_c1_g2 | 1.883 | 5.602 | 4.881 | 0.000 | 0.000 | 0.000 | 0.000 |
| TRINITY_DN37146_c4_g1 | 3.707 | 6.620 | 2.403 | 0.000 | 0.000 | 0.000 | 0.000 |
| TRINITY_DN106308_c0_g1 | 5.307 | 4.685 | 3.443 | 0.000 | 0.000 | 0.000 | 0.000 |
| TRINITY_DN15779_c0_g4 | 2.524 | 4.491 | 1.637 | 0.000 | 0.000 | 0.000 | 0.000 |
| TRINITY_DN72281_c1_g1 | 0.518 | 17.904 | 3.031 | 0.000 | 0.000 | 0.000 | 0.000 |
| TRINITY_DN35940_c2_g1 | 4.097 | 16.138 | 0.441 | 0.000 | 0.000 | 0.000 | 0.000 |
| TRINITY_DN96807_c0_g1 | 2.624 | 3.111 | 3.406 | 0.000 | 0.000 | 0.000 | 0.000 |
| TRINITY_DN13826_c1_g1 | 2.573 | 4.577 | 1.669 | 0.000 | 0.000 | 0.000 | 0.000 |
| TRINITY_DN26809_c1_g1 | 3.815 | 1.317 | 4.317 | 0.000 | 0.000 | 0.000 | 0.000 |
| TRINITY_DN46948_c2_g1 | 4.079 | 6.319 | 1.322 | 0.000 | 0.000 | 0.000 | 0.000 |
| TRINITY_DN5843_c11_g1 | 5.018 | 1.991 | 2.167 | 0.000 | 0.000 | 0.000 | 0.000 |
| TRINITY_DN66551_c0_g1 | 4.770 | 6.596 | 6.186 | 0.000 | 0.000 | 0.000 | 0.000 |
| TRINITY_DN10275_c1_g1 | 14.992 | 1.165 | 7.029 | 0.000 | 0.000 | 0.000 | 0.000 |
| TRINITY_DN4319_c1_g1 | 3.110 | 3.676 | 2.019 | 0.000 | 0.000 | 0.000 | 0.000 |
| TRINITY_DN3028_c5_g1 | 9.539 | 5.702 | 3.273 | 0.000 | 0.000 | 0.000 | 0.000 |
| TRINITY_DN4147_c19_g1 | 6.929 | 2.729 | 1.452 | 0.000 | 0.000 | 0.000 | 0.000 |
| TRINITY_DN47093_c0_g1 | 3.298 | 4.311 | 1.211 | 0.000 | 0.000 | 0.000 | 0.000 |
| TRINITY_DN19947_c0_g2 | 1.220 | 9.410 | 1.578 | 0.000 | 0.000 | 0.000 | 0.000 |
| TRINITY_DN27447_c3_g1 | 2.557 | 3.788 | 3.304 | 0.000 | 0.000 | 0.000 | 0.000 |
| TRINITY_DN196927_c0_g1 | 4.079 | 6.062 | 1.322 | 0.000 | 0.000 | 0.000 | 0.000 |
| TRINITY_DN77912_c0_g1 | 9.491 | 0.941 | 13.524 | 0.000 | 0.000 | 0.000 | 0.000 |
| TRINITY_DN54191_c1_g1 | 1.843 | 6.470 | 2.106 | 0.000 | 0.000 | 0.000 | 0.000 |
| TRINITY_DN23681_c0_g1 | 4.079 | 4.850 | 3.965 | 0.000 | 0.000 | 0.000 | 0.000 |
| TRINITY_DN5547_c4_g1 | 3.886 | 3.468 | 2.518 | 0.000 | 0.000 | 0.000 | 0.000 |
| TRINITY_DN61499_c0_g1 | 3.596 | 2.141 | 2.331 | 0.000 | 0.000 | 0.000 | 0.000 |
| TRINITY_DN13061_c2_g1 | 3.443 | 4.099 | 2.230 | 0.000 | 0.000 | 0.000 | 0.000 |
| TRINITY_DN82594_c0_g1 | 3.058 | 3.351 | 1.703 | 0.000 | 0.000 | 0.000 | 0.000 |
| TRINITY_DN192471_c0_g1 | 8.246 | 2.944 | 4.272 | 0.000 | 0.000 | 0.000 | 0.000 |
| TRINITY_DN95919_c0_g1 | 7.023 | 1.390 | 3.037 | 0.000 | 0.000 | 0.000 | 0.000 |
| TRINITY_DN26527_c1_g1 | 7.023 | 2.779 | 3.037 | 0.000 | 0.000 | 0.000 | 0.000 |
| TRINITY_DN204292_c0_g1 | 4.436 | 2.634 | 1.439 | 0.000 | 0.000 | 0.000 | 0.000 |
| TRINITY_DN174129_c0_g1 | 7.258 | 1.469 | 3.211 | 0.000 | 0.000 | 0.000 | 0.000 |
| TRINITY_DN850_c72_g1 | 2.524 | 4.491 | 6.547 | 0.000 | 0.000 | 0.000 | 0.000 |
| TRINITY_DN39963_c1_g1 | 30.170 | 43.253 | 35.481 | 5.068 | 3.939 | 5.346 | 9.257 |
| TRINITY_DN43705_c0_g1 | 259.679 | 66.811 | 269.017 | 0.000 | 0.669 | 0.573 | 1.037 |
