# Supplementary material for: First neurotranscriptome of adults Tambaquis (Colossoma macropomum) with characterization and differential expression between males and females
Source: Sci Rep. 2024 Feb 7;14:3130. doi: 10.1038/s41598-024-53734-5 (PMC10850070; doi:10.1038/s41598-024-53734-5)
Supplement: Supplementary file 5 — Supplementary Table S2. [file 41598_2024_53734_MOESM5_ESM.docx]

**Table S2:** Functional annotation of differentially expressed transcripts in the neurotranscriptome of Tambaqui based on Blast2Go searches.

| Description | Sex | Length | Species | #Hits | e-Value | Mean similarity |
| --- | --- | --- | --- | --- | --- | --- |
| **GAPDHS** - Glyceraldehyde-3-Phosphate dehydrogenase 2 | Female | 340 | *Danio rerio* | 5 | 1.63E-45 | 84.5 |
| **SUPT6H** - Histone chaperone and transcription elongation factor | Female | 216 | *Danio rerio* | 5 | 5.77E-33 | 82.88 |
| **UCHL5** - Ubiquitin carboxyl-terminal hydrolase 5 | Female | 280 | *Mus musculus* | 5 | 1.86E-18 | 69.53 |
| **ZFP54** - Zinc finger protein 54 | Female | 234 | *Mus musculus* | 5 | 9.23E-25 | 80.0 |
| **RNH1** – Ribonuclease/angiogenin inhibitor 1 | Female | 293 | *Sus scrofa* | 5 | 4.59E-12 | 66.88 |
| **SLC2A6** - Solute carrier family 2, member 6 | Female | 576 | *Homo sapiens* | 5 | 3.98E-04 | 80.5 |
| **ZSCAN20** - Zinc finger and SCAN domain containing 20 | Female | 234 | *Homo sapiens* | 5 | 1.73E-22 | 75.06 |
| **TC3 Transposase** - HTH_Tnp_Tc3_2 domain | Female | 442 | *Seriola dumerili* | 5 | 6.82E-44 | 71.79 |
| **AHNAK** - Neuroblast differentiation-associated protein | Female | 203 |  | 5 | 1.34E-07 | 66.95 |
| **NXF1 -** Nuclear RNA export factor 1 | Female | 220 |  | 3 | 2.30E-02 | 49.74 |
| **TCB1 -** Transposable element | Female | 211 |  | 2 | 1.74E-08 | 78.26 |
| **4aOHBH_4_** **-** 4a-hydroxytetrahydrobiopterin dehydratase | Female | 318 | *Pygocentrus nattereri* | 1 | 4.96E+01 | 67.65 |
| **NLRC3** - NLR family CARD domain-containing protein 3 | Female | 360 |  | 5 | 4.91E-40 | 76.95 |
| **NLRP3** - NACHT, LRR and PYD domains-containing protein 3 | Female | 211 | *Sinocyclocheilus grahami* | 5 | 4.87E-27 | 79.43 |
| **GRIN1** - Glutamate ionotropic receptor NMDA type subunit 1 | Female | 321 | *Pygocentrus nattereri* | 2 | 3.57E-112 | 91.46 |
| **ZDHHC3** – Zinc finger DHHC-typePalmitoyl transferase | Female | 209 | *Colossoma macropomum* | 2 | 2.29E-77 | 94.26 |
| **SERINC1** - Serine incorporator 1 | Female | 202 | *Pygocentrus nattereri* | 5 | 1.43E-19 | 94.01 |
| **SRRM3** - Serine/arginine repetitive matrix 3 | Female | 269 | *Colossoma macropomum* | 2 | 9.05E-33 | 85.76 |
| **TCEANC** - Transcription elongation factor A N-terminal and central domain containing | Female | 204 | *Pygocentrus nattereri* | 3 | 2.24E-72 | 87.29 |
| **RPRMA** - Protein reprimo A | Female | 217 | *Pygocentrus nattereri* | 4 | 3.40E-11 | 91.63 |
| **SNX10** - Sorting nexin 10 | Female | 208 | *Homo sapiens* | 5 | 1.45E-34 | 93.14 |
| **OLPB** - Cell surface glycoprotein 1 | Female | 444 | *Colossoma macropomum* | 1 | 2.23E-150 | 97.55 |
| **BSG** - Basigin | Female | 233 | *Colossoma macropomum* | 2 | 3.34E-83 | 91.91 |
| **LRCH1** - Leucine rich repeats and calponin homology domain containing 1 | Female | 215 | *Pygocentrus nattereri* | 5 | 3.34E-16 | 81.17 |
| **MDM2** - E3 ubiquitin-protein ligase | Female | 254 | *Callithrix jacchus* | 5 | 1.77E-59 | 88.93 |
| **IgC MHC I alpha3** - BOLA Class I histocompatibility antigen, alpha chain BL3-7 | Female | 250 | *Pygocentrus nattereri* | 2 | 6.47E-35 | 84.48 |
| **TYMP** - Thymidine phosphorylase | Female | 216 | *Pygocentrus nattereri* | 1 | 3.35E-16 | 80.77 |
| **VANGL2** - VANGL planar cell polarity protein 2 | Female | 216 | *Pygocentrus nattereri* | 1 | 5.30E-54 | 96.55 |
| **TAGLN** - Transgelin | Female | 234 | *Astyanax mexicanus* | 1 | 6.12E-19 | 88.35 |
| **GTF2H5** - General transcription factor IIH subunit 5 | Female | 201 | *Pygocentrus nattereri* | 5 | 6.71E-08 | 87.82 |
| **ACKR3** - Atypical chemokine receptor 3 | Female | 226 | *Callithrix jacchus* | 5 | 5.29E-94 | 95.57 |
| **CEPT1** - Choline/ethanolamine phosphotransferase 1 | Female | 231 | *Pygocentrus nattereri* | 5 | 2.20E-08 | 92.5 |
| **GIMAP7** - Gtpase IMAP family member 7 | Female | 232 | *Pygocentrus nattereri* | 5 | 1.28E-35 | 89.87 |
| **SKAP2** - Src kinase associated phosphoprotein 2 | Female | 229 | *Colossoma macropomum* | 1 | 2.18E-08 | 100.0 |
| **ARRDC1B** - Arrestin domain containing 1b | Female | 208 | *Colossoma macropomum* | 1 | 2.50E-12 | 86.17 |
| **PRPF39** - Pre-mRNA-processing factor 39 | Female | 204 | *Pygocentrus nattereri* | 2 | 1.13E-15 | 81.58 |
| **NUDT15** - Nudix hydrolase 15 | Female | 235 | *Pygocentrus nattereri* | 2 | 6.24E-10 | 89.22 |
| **COQ4** - Coenzyme Q4 | Female | 230 | *Pygocentrus nattereri* | 1 | 2.17E-13 | 95.38 |
| **HA1F** - Class I histocompatibility antigen, F10 alpha chain | Female | 254 | *Colossoma macropomum* | 5 | 2.72E-117 | 95.91 |
| **NDUFB5** - NADH: ubiquinone oxidoreductase subunit B5 | Female | 211 | *Sapajus apella* | 5 | 1.40E-69 | 92.17 |
| **FUCL4** - Fucolectin-4 | Male | 1481 | *Anguilla japonica* | 5 | 2.09E-36 | 65.26 |
| **CNTN1** - Contactin-1 | Male | 409 | *Gallus gallus* | 4 | 6.81E-01 | 71.82 |
| **ZNF479** - Zinc finger protein 479 | Male | 204 | *Homo sapiens* | 5 | 1.48E-15 | 73.94 |
| **CYP1A1** - Cytochrome P450 | Male | 261 | *Oryctolagus cuniculus* | 5 | 5.70E-06 | 62.3 |
| **GPR1** - G_PROTEIN_RECEP_F1_2 domain-containing protein | Male | 210 | *Pygocentrus nattereri* | 1 | 1.19E-01 | 58.14 |
| **FIG4** - Phosphoinositide 5-phosphatase | Male | 220 | *Pygocentrus nattereri* | 5 | 5.66E-03 | 64.79 |
| **PKA -** Protein kinase domain-containing protein | Male | 345 | *Salmo trutta* | 5 | 4.68E-11 | 62.61 |
| **NACHT** domain - containing protein | Male | 420 | *Salmo trutta* | 4 | 1.10E-03 | 51.13 |
| **PBXIP1** - Pre-B-cell leukemia transcription factor-interacting protein 1 | Male | 232 | *Pygocentrus nattereri* | 2 | 1.27E-40 | 92.2 |
| **MKNK1** - MAP kinase interacting serine/threonine kinase 1 | Male | 214 | *Pygocentrus nattereri* | 5 | 3.39E-01 | 96.31 |
| **KIF3C** - Kinesin-like protein | Male | 212 | *Pygocentrus nattereri* | 5 | 1.16E-25 | 94.29 |
| **SPSB4** - SPRY domain-containing SOCS box protein 4 | Male | 653 | *Pygocentrus nattereri* | 4 | 4.12E-10 | 94.97 |
| **CCDC92** - Coiled-coil domain containing 53 | Male | 224 | *Pygocentrus nattereri* | 1 | 1.66E+01 | 76.92 |
| **AKAP17A** - A kinase anchor protein 17A | Male | 216 | *Colossoma macropomum* | 1 | 1.55E-19 | 80.5 |
| **NEK11** - NIMA related kinase 11 | Male | 203 | *Pygocentrus nattereri* | 1 | 1.47E-04 | 84.21 |
| **SMOC1** - SPARC related modular calcium binding 1 | Male | 206 | *Pygocentrus nattereri* | 5 | 4.13E-11 | 89.33 |
| **CAMKMT** - Calmodulin-lysine N-methyltransferase | Male | 232 | *Pygocentrus nattereri* | 3 | 1.25E-50 | 83.84 |
| **KCNC1** - Potassium voltage-gated channel subfamily C member 1 | Male | 220 | *Pygocentrus nattereri* | 2 | 5.82E-04 | 93.06 |
| **AAK1** - AP2 associated kinase 1a | Male | 237 | *Colossoma macropomum* | 4 | 6.34E-04 | 90.2 |
| **TRPC4** - Short transient receptor potential channel 4 | Male | 283 | *Pygocentrus nattereri* | 1 | 9.86E-18 | 98.51 |
